# Consequences of Hypoxic Events, Necrosis, and Microvascular Density, in Astrocytoma IDH-Mutant, CNS WHO Grade 4

**DOI:** 10.3390/medsci14010006

**Published:** 2025-12-23

**Authors:** Cristian Ionut Orasanu, Madalina Bosoteanu, Sorin Vamesu, Raluca Ioana Voda, Anamaria Sincu, Mariana Deacu

**Affiliations:** 1 Department of Pathology, Clinical Service of Pathology, “Sf. Apostol Andrei” Emergency County Hospital, 900591 Constanta, Romania; mbosoteanu@yahoo.com (M.B.); sorinvamesu@yahoo.com (S.V.); anamariasincu5@gmail.com (A.S.); deacu_mariana@yahoo.com (M.D.); 2 Center for Research and Development of the Morphological and Genetic Studies of Malignant Pathology (CEDMOG), “Ovidius” University, 900591 Constanta, Romania; raluca.v1694@yahoo.ro; 3 Faculty of Medicine, “Ovidius” University of Constanta, 900470 Constanta, Romania

**Keywords:** astrocytoma, necrosis, microvascularisation, risk

## Abstract

**Background/Objectives:** Astrocytoma IDH-mutant CNS WHO grade 4 is a malignant tumor of the central nervous system characterized by tumor necrosis, microvascular proliferation, and/or homozygous CDKN2A/B deletion. This study aims to investigate the prognostic role of the consequences of hypoxic events leading to necrosis and microvascular density, observing their associations with clinical-imaging parameters and morphogenetics. **Methods:** We performed a retrospective analysis over a 10-year period. Clinical and imaging data were collected from observation sheets and electronic databases. Six immunohistochemical markers and FISH testing were used to evaluate the prognosis and neoformation of blood vessels. Based on the whole slide image, the necrotic percentage was assessed, and the microvascular density was quantified. All data were statistically analyzed. **Results:** We identified 44 cases, with a mean age of 57.86 years. From a clinical perspective, advanced age, arterial hypertension, diabetes mellitus, and acute onset of clinical manifestations represent negative prognostic factors. In imaging, the increased rate of resectability is a protective factor, while the presence of residual volume and an increased residual volume have a negative impact on survival. The consequences of hypoxic events (tumor necrosis and microvascular density) are negative risk factors for survival. Added to these are p53 overexpression, loss of PTEN, deletion, and amplification of the CDKN2A gene. **Conclusions:** We observed that necrosis and increased microvascular density resulting from microvascular proliferation are both defining features of the tumor and impact patient prognosis and survival. In addition, they induce or are associated with other essential changes (p53, PTEN, CDKN2A) that promote tumor aggressiveness.

## 1. Introduction

Astrocytoma IDH-mutant, CNS WHO grade 4 (A4), is classified as a high-grade malignant brain tumor by the World Health Organization (WHO) [1]. It is a diffuse glioma of adults, with a predominant supratentorial location. It is found mainly in patients in the fourth and fifth decades of life. Its defining characteristics are the presence of tumor necrosis, microvascular proliferation, and/or homozygous deletion of Cyclin-Dependent Kinase Inhibitor 2A/B (CDKN2A/B) [2,3].

Hypoxia contributes to a decrease in pH, leading to necrosis and activation of anaerobic glycolysis-based metabolism. Tumor necrosis, the defining feature of A4, is more common in tumors larger than 4 mm. The onset of necrosis occurs in the central area of the tumor, as this area is less vascularized and nutrient supply is reduced [4,5]. Tumor necrosis can present in various forms. The characteristic form of A4 is called “pseudopalisading”. This microscopic appearance is characterized by densely packed areas of tumor cells arranged radially around foci of necrosis. These cells express increased Hypoxia-Inducible Factor (HIF), leading to an exuberant angiogenic response [6,7].

Angiogenesis is an essential factor in tumor growth. Proangiogenic factors, such as Vascular Endothelial Growth Factor (VEGF), are a consequence of hypoxia, stimulating microvascular proliferation and neoangiogenesis. VEGF is a pro-angiogenic factor by stimulating endothelial cell proliferation, migration, and mitotic stimulation of these cells, resulting in the proliferation of new vessels with a role in tumor progression [4,8]. Four patterns of vascular proliferation have been identified, such as microvascular sprouting, microvascular clustering, vascular garland, and glomeruloid tuft. Microvascular sprouting is characterized by the presence of small capillary-like vessels, distributed diffusely and uniformly throughout the vital tumor tissue. Microvascular clustering is characterized by small vessels arranged in groups (minimum 3 vessels) without the presence of stroma between them [9,10]. A vascular garland is a group of small vessels arranged in a garland-like manner. Intervening conjunctival stroma may or may not be present. This vascular pattern is particularly common around necrotic tissue. A glomeruloid tuft is a group of vessels with intervening stroma, in which pericytes and endothelial cells form dysfunctional, poorly organized structures with an architecture similar to renal glomeruli [9,10,11].

In a previous study, we analyzed cellular immaturity and the consequences of hypoxia in a group of glioblastoma IDH wild-type WHO grade 4, where we did not identify tumor necrosis or microvascular density as independent risk factors for survival [12]. Because it is a recent entity with ancient roots, few studies have focused solely on this tumor type. The presence of microvascularization and necrosis processes are defining elements, but they must be investigated, not only in the pathogenic process, but also as an impact on the evolution of the case, the patient’s prognosis, and possibly as a therapeutic target. Therefore, this study seeks to investigate the prognostic role of hypoxic events that lead to necrosis and increased microvascular density in patients diagnosed with A4 while examining their associations with clinical and morphogenetic imaging parameters.

## 2. Materials and Methods

**Figure 1:**
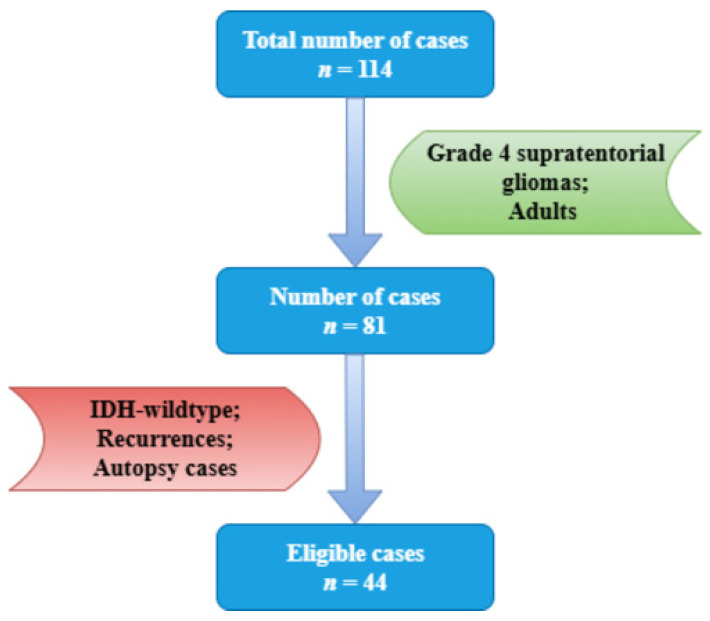
Case selection according to inclusion and exclusion criteria.

A retrospective study was conducted over a 10-year period, involving patients diagnosed with primary central nervous system tumors admitted to the County Emergency Clinical Hospital of Constanta. The inclusion criteria comprised a histopathological and immunohistochemical diagnosis of grade 4 supratentorial gliomas, following the latest WHO criteria (2021), and adult patients. It is worth mentioning that at the time of diagnosis, the cases were tested for IDH mutation and ATRX retention/loss. Exclusion criteria consisted of IDH-wildtype status, recurrences, and cases diagnosed at autopsy (Figure 1).

Imaging examinations were performed before neurosurgical intervention and focused on tumor location, diameter, and volume, peritumoral edema, and midline shift. The type of total or subtotal neurosurgical ablation was evaluated postoperatively. The resectability rate was automatically calculated based on the remaining tumor volume.

**Figure 2:**
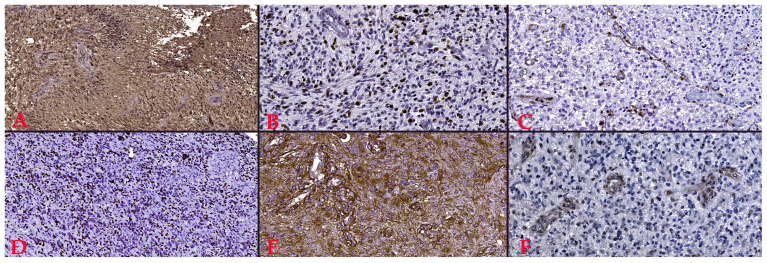
(**A**). Positive reaction to IDH1 (R132H), Ob. x100. (**B**). Proliferative index of 40%, Ki-67 staining, Ob. x200. (**C**). Lost PTEN reaction, Ob. x200. (**D**). Overexpression of p53, Ob. x100. (**E**). Intensely positive reaction to Nestin, Ob. x200. (**F**). 10–50% positive reaction to MGMT, Ob. x200.

**Figure 3:**
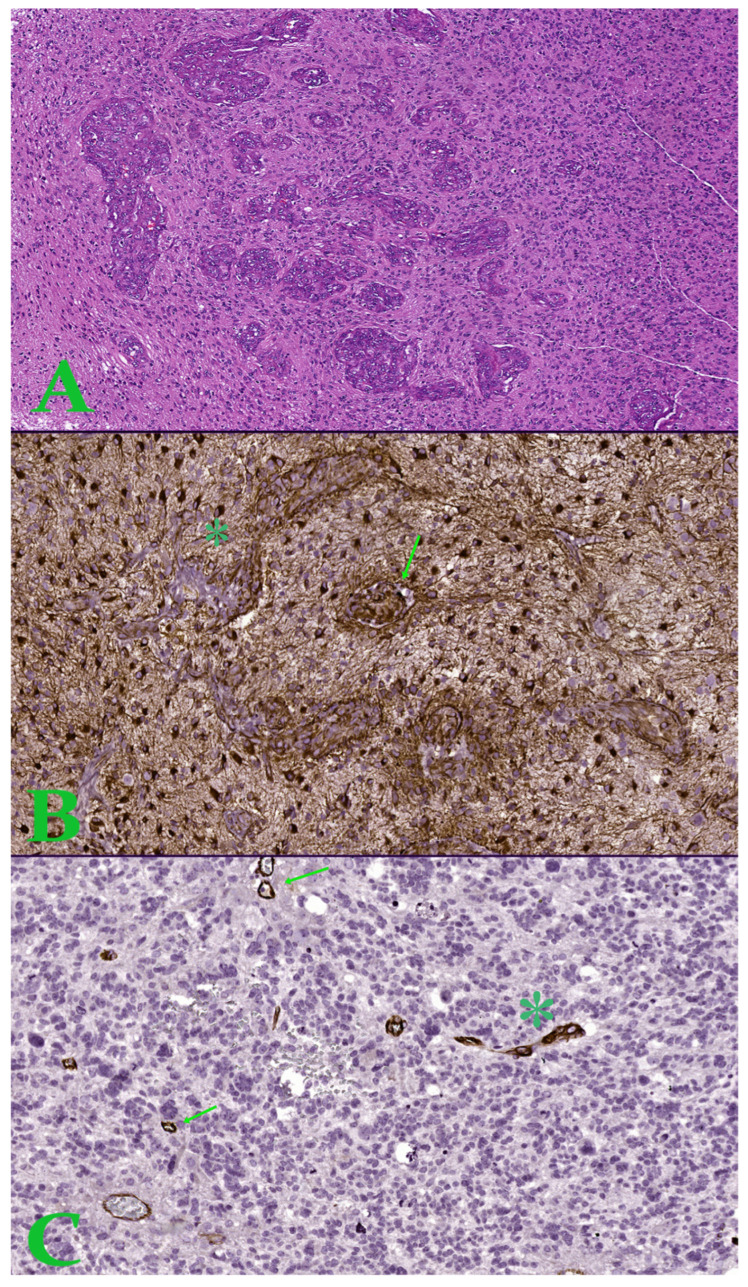
(**A**). Image in hematoxylin-eosin, which shows an area of microvascular proliferation. Ob. x50. (**B**). Hot-spot of 1 mm^2^ with increased microvascular density showing proliferation of glomeruloid tuft type (arrow) and vascular garland (asterisk)—Nestin stain. (**C**). Hot-spot of 1 mm^2^ with increased microvascular density showing microvascular sprouting (arrow) and microvascular clustering (asterisk)—Nestin stain.

**Figure 4:**
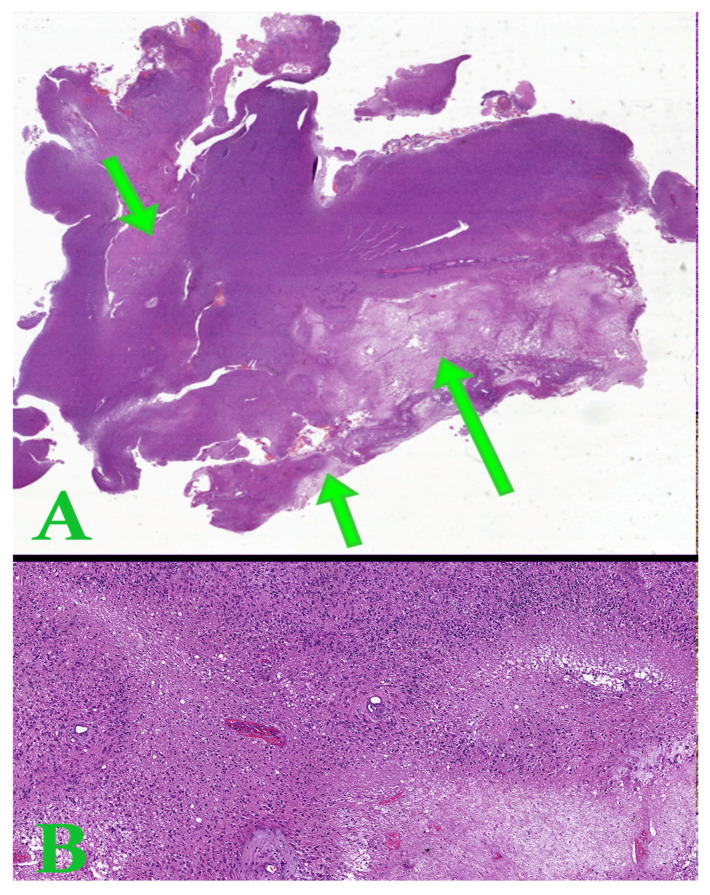
(**A**). Whole slide image representing a tumor section of astrocytoma with areas of tumor necrosis (arrows). (**B**). Hematoxylin-eosin image with pseudopalisade necrosis. Ob. x50.

Six immunohistochemical markers were performed within CEDMOG. Immunohistochemical assays were performed by the HIER-DAB method and employed the following markers: IDH1 R132H (clone H09), Ki-67 (clone SP6), PTEN (phosphatase and tensin homolog, clone 6H2.1), p53 (clone SP5), Nestin (clone 10C2), and MGMT (clone MT 23.2) (Figure 2). The stratification of cases was due to the IDH1 R132H immunomarker. The Ki-67 index was determined by calculating the percentage of positive nuclei, based on the assessment of a minimum of 10 high-power fields, totaling at least 1000 nuclei. The PTEN was assessed at both the cytoplasmic and nuclear levels. An expression above 10% was considered positive for p53 [13,14]. The assessment of Nestin involved evaluating the cytoplasmic immunoreactivity of glial cells across three levels (weak, moderate, or strong) and determining the reactivity (negative or positive) in endothelial cells (Figure 3). Slides were converted to digital format using a TissueScope LE120 Slide Scanner (Huron Digital Pathology, Ontario, CA, USA). Following that, were identified ten microvascular hotspot regions, each measuring 1 mm^2^. The average count of capillary vessels assessed by two pathologists was divided by ten to yield the mean number of vessels per 1 mm^2^. To quantify necrosis, the tumor section was divided into 100 squares. Where necrosis was present, the square was shaded. The sum of the shaded squares represents the necrotic percentage (Figure 4). The nuclear level of MGMT was evaluated in glial tumor cells, and the results were subsequently categorized as >50%, 10–50%, and <10%.

The CDKN2A gene was examined through fluorescent in situ hybridization (FISH). ZytoLight SPEC CDKN2A/CEN 9 Dual Color Probe probes (Bremerhaven, Bremen, Germany) were employed for the cytogenetic evaluation. The Zeiss Axio Imager 2 microscope (Zeiss Gmbh, Jena, Germany) was employed to compute the fluorescent signals in 100 tumor nuclei. Two green signals (CDKN2A gene region) and two orange signals (CEN 9 probe) were detected in normal cells. In the presence of deletions, there were fewer green signals, while amplifications resulted in a greater number of green signals. A homozygous deletion means the absence of green signals and the presence of at least one orange signal in the nucleus.

A statistical data analysis was performed in SPSS Statistics Version 26 (IBM Corporation, Armonk, NY, USA). Indicators of central tendency and variability were used. Univariate data analysis was performed using the chi-square test or Fisher’s exact test for categorical data, and the Mann–Whitney U test and the Kruskal–Wallis H test were performed for continuous variables. We applied the Pearson Correlation Coefficient to ascertain the relationship of the data. The accuracy of the parameters was determined by calculating the receiver operating characteristic (ROC) as well as the area under the curve (AUC) values. The optimal cut-off point for the parameters is defined by their sensitivity and specificity, which maximizes the AUC. The Kaplan–Meier was employed to calculate survival estimates over a five-year follow-up period. The log-rank test was implemented to evaluate the survival disparities between the categories. Hazard ratios (HR) were estimated using univariate and multivariate Cox regression analysis. A *p*-value of less than 0.05 was used to determine statistical significance.

The ethics opinion was obtained from the local ethics commission (Ethics Commission of the Constanta County Emergency Hospital; No. 04; Approval date: 4 February 2022), and all patients endorsed the informed consent form at the time of hospitalization.

## 3. Results

### 3.1. Demographic and Clinical Aspects

After applying the inclusion and exclusion criteria, we identified 44 cases of A4. Most patients were identified in the 5th and 6th decades of life (29.55% and 27.27%, respectively). The mean age at diagnosis was 57.86 years, with 34.09% being over 65 years. We observed a slight male predominance of 54.55%.

Over half of the patients had an acute onset of symptoms, less than two weeks before presenting to the doctor (54.55%). The most frequent clinical manifestations were motor deficits (65.91%), headache (47.73%), and cognitive disorders (43.18%). The most common associated comorbidities were diabetes mellitus (29.55%) and arterial hypertension (27.27%).

**Figure 5:**
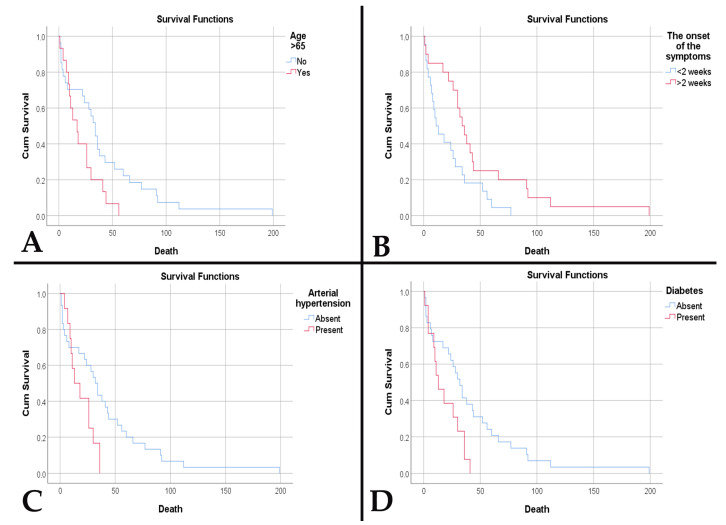
(**A**). Kaplan–Meier survival plot shows a lower survival rate for patients older than 65 years (*p* = 0.038). (**B**). Kaplan–Meier survival plot shows a lower survival rate for patients with an acute onset of symptoms (*p* = 0.017). (**C**). Kaplan–Meier survival plot shows a lower survival rate for patients with arterial hypertension (*p* = 0.023). (**D**). Kaplan–Meier survival plot shows a lower survival rate for patients with diabetes mellitus (*p* = 0.019).

Regarding survival, we observed a lower survival in the decades of the extremities included in the study: 2 weeks for the fourth decade and 5.5 weeks for the ninth decade (*p* < 0.001). Age at diagnosis was a risk factor for death (HR = 1.035, *p* = 0.022). In the case of patients over 65 years of age, survival was lower (20.87 weeks vs. 42 weeks, *p* = 0.038) (Figure 5A). Age over 65 years was an independent risk factor for death (HR = 2.023, *p* = 0.044). Furthermore, acute onset of symptoms was associated with lower survival (22.14 weeks vs. 48 weeks, *p* = 0.017) (Figure 5B) and was an independent risk factor for death (HR = 2.173, *p* = 0.020). Patients with diabetes and hypertension had a lower survival (18.39 weeks vs. 41.66 weeks, *p* = 0.019, and 18.83 weeks vs. 40.7 weeks, *p* = 0.023, respectively) (Figure 5C,D). Thus, diabetes and hypertension represented negative risk factors for survival (HR = 2.311, *p* = 0.024, and HR = 2.325, *p* = 0.029, respectively).

### 3.2. Imaging Aspects

We observed a similar distribution of lesions across the cerebral hemispheres, with a predominance over the temporal (25%), frontal (18.18%), and fronto-parietal (18.18%) lobes. The mean maximum diameter and mean tumor volume identified were 50.39 mm and 86.14 cm^3^, respectively. These dimensions produced a mean midline shift of 8.91 mm, with peritumoral edema present in 90.91% of cases. Complete surgical excision was observed in 77.27% of cases. An average resection rate of 87.67% was observed, with a mean tumor volume of 11.02 cm^3^. Complete trimodal treatment was achieved in 68.18% of cases. At the end of the study, 95.45% of patients were declared deceased. The mean survival was 34.45 weeks.

A statistically significant association was observed between the affected hemisphere and symptoms. Thus, the presence of epilepsy correlated with the localization in the right hemisphere (*p* = 0.008), and cognitive disorders with the presence in the left hemisphere (*p* = 0.034). Increased maximum tumor diameter was associated with the presence of peritumoral edema (*p* = 0.004) and an increased residual volume (*p* < 0.001). A maximum diameter ≥ 35 mm is predictive for the occurrence of peritumoral edema (sensitivity 87.5% and specificity 100%, AUC = 0.913, *p* = 0.007).

**Figure 6:**
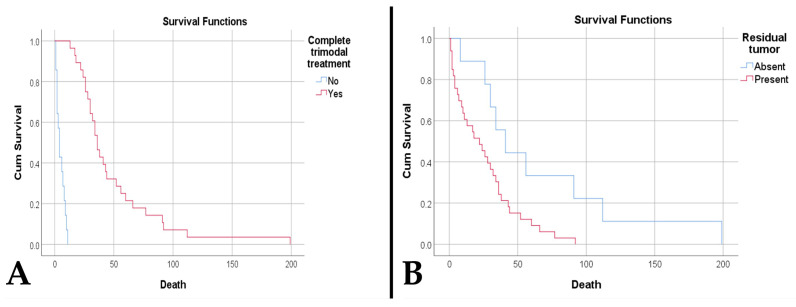
(**A**). Kaplan–Meier survival plot shows a lower survival rate for patients who did not receive the complete trimodality treatment (*p* < 0.001). (**B**). Kaplan–Meier survival plot shows a lower survival rate for patients who had residual tumor (*p* = 0.017).

The survival rate was significantly higher among patients who received complete trimodal treatment (49.18 weeks compared to 5 weeks, *p* < 0.001). (Figure 6A). The presence of tumor residue was associated with poor survival (25.76 weeks vs. 66.33 weeks, *p* = 0.017) (Figure 6B). The presence of tumor residue along with an increased residual volume represents negative risk factors, while an increased resectability rate is a protective factor (Table 1).

### 3.3. Morphogenetic Aspects

The main histopathological aspects and genetic analysis of the CDKN2A gene were presented in Table 2.

An increased proliferation index was correlated with MGMT > 50% (*p* < 0.001) and a negative/weakly positive nestin immunoreaction (*p* = 0.003). A Ki67 index ≥ 55% is predictive of an MGMT above 50% (sensitivity 100%, specificity 92.9%, AUC 0.980, *p* < 0.001), and a Ki67 ≤ 27.5% is predictive of an MGMT < 10% (sensitivity 84.6%, specificity 90.3%, AUC = 0.876, *p* < 0.001). MGMT > 50% was associated with a negative/weakly positive nestin immunoreaction (*p* = 0.002). Overexpression of p53 was associated with an increased residual volume (*p* = 0.041) and a reduced resectability rate (*p* = 0.035). A similar aspect was observed in the loss of PTEN immunoexpression (*p* = 0.014 and *p* = 0.001, respectively). Preserved PTEN correlated with complete trimodal treatment (*p* < 0.001).

A higher percentage of tumor necrosis correlated with the presence of diabetes mellitus (*p* = 0.038), increased peritumoral edema (*p* = 0.032), a diminished resection rate (*p* < 0.001), and a higher residual volume (*p* < 0.001). A percentage ≥ 19.5% is predictive of peritumoral edema (sensitivity 82.5%, specificity 75%, AUC = 0.847, *p* = 0.023). Furthermore, increased tumor necrosis percentage was associated with a directly proportional increase in MGMT immunoreaction (*p* = 0.022), p53 overexpression (*p* = 0.036), and PTEN loss (*p* < 0.001). A percentage ≥ 31% is predictive of MGMT > 50% (sensitivity 68.8%, specificity 57.1% AUC = 0.692, *p* = 0.036) and PTEN loss (sensitivity 100%, specificity 65.6%, AUC = 0.874, *p* < 0.001). A percentage of ≥24.5% necrosis is predictive of p53 overexpression (sensitivity 74.3%, specificity 66.7%, AUC = 0.741, *p* = 0.027).

Elevated microvascular density correlated with a heightened necrotic percentage (*p* < 0.001), p53 overexpression (*p* = 0.047), a directly proportional increase in MGMT immunointensity (*p* = 0.027), and PTEN loss (*p* = 0.002). Thus, a microvascular density ≥ 51.7 vessels/mm^2^ is predictive of PTEN loss (sensitivity 83.3%, specificity 62.5%, AUC = 0.789, *p* = 0.003). Increased microvascular density correlated with age over 65 years (*p* = 0.015), female gender (*p* = 0.045), presence of diabetes mellitus (*p* = 0.043), presence of peritumoral edema (*p* = 0.020), reduced resectability rate (*p* < 0.001), increased maximum tumor diameter (*p* = 0.013), increased residual volume (*p* < 0.001), and absence of complete treatment (*p* < 0.001). A microvascular density ≥ 31.8 vessels/mm^2^ is predictive of the occurrence of peritumoral edema (sensitivity 95%, specificity 75%, AUC = 0.813, *p* = 0.041).

Both microvascular density and necrotic percentage correlated with gene amplifications, followed by their deletions (*p* = 0.003, *p* = 0.008, respectively). Also, the resectability rate was lower in amplifications and deletions (*p* = 0.045). Normal gene status was associated with normal p53 (*p* = 0.045). In the case of deletions, correlations were observed with MGMT > 50% (*p* = 0.016).

**Figure 7:**
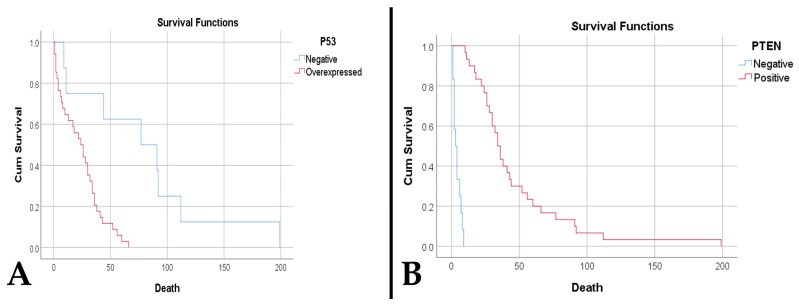
(**A**). Kaplan–Meier survival plot shows a lower survival rate for cases with p53 overexpression (*p* < 0.001). (**B**). A. Kaplan–Meier survival plot shows a lower survival rate for cases with PTEN loss (*p* < 0.001).

Regarding survival, we observed that an increased necrotic percentage was associated with a reduced time to death (*p* < 0.001). In addition, an elevated microvascular density was associated with both a reduced time to death (*p* < 0.001) and death (*p* = 0.017). Thus, a density ≥ 32.15 vessels/mm^2^ is predictive of death (sensitivity 90.5%, specificity 100%, AUC = 0.952, *p* = 0.032). Also, a decreased survival was observed in patients with overexpressed p53 (23.88 weeks vs. 79.38 weeks, *p* < 0.001) (Figure 7A), absent PTEN (4.08 weeks vs. 46.6 weeks, *p* < 0.001) (Figure 7B), amplifications and deletions of the CDKN2A gene (9.67 weeks vs. 22.7 weeks vs. 50.74 weeks, *p* = 0.002).

Univariate analysis identified as risk factors for death MGMT 10–50%, overexpressed p53, PTEN loss, an increased percentage of tumor necrosis, an increased microvascular density, deletion and amplification of the CDKN2A gene (Table 3).

## 4. Discussion

Astrocytoma IDH-mutant CNS WHO grade 4 does not show a predilection for age; it can occur at any time. However, it was observed that most cases were in patients under 55 years of age, with a median of 38 years [15]. We observed an older age, with a median of 56 years. The literature notes that individuals over 65 years of age rarely have IDH mutations. Advanced age represents a poor prognosis for gliomas. These aspects should not influence the therapeutic decision or, worse, cast doubt on the diagnosis, redirecting the clinician to a wildtype status [16]. So far, it is certain that in astrocytomas IDH-mutant CNS WHO grades 2 and 3, low age was associated with increased survival. However, more consideration should be given to the molecular aspect regarding life expectancy [17]. While the age situation in A4 remains uncertain, age is a stated risk factor for survival in glioblastomas [18]. This is one of the first studies after the new classification in which the role of advanced age in patient survival is directly observed.

As in the study by Miller et al., who reported a higher frequency of these tumors among males (1.3:1), we observed the same aspect [19]. Unlike the wildtype IDH status counterpart, where females show better survival, this aspect was not observed in the case of mutant IDH status [20].

The symptomatology is nonspecific. It can be easily confused with a stroke or inflammatory lesions. The difference is the onset of symptoms and presentation to the doctor [21]. We have not identified studies that analyze the period between the onset of symptoms and presentation to the doctor in the case of A4. This is one of the first studies to highlight this aspect and identify acute presentation of symptoms as a negative risk factor.

Clinical manifestations are caused by three mechanisms: tumor location, increased intracranial pressure, and the direct effect of tumor necrosis. Depending on the tumor topography, 20–40% of cases may present with partial or generalized epileptic seizures. The increase in the size of the process determines peritumoral edema with increased intracranial hypertension. This syndrome has as its main manifestation headache, usually unilateral, with progressive severity. The presence of tumor tissue destruction, i.e., tumor necrosis, is responsible for the appearance of focal neural deficits and cognitive deficits [21,22]. In the present study, we observed a higher frequency of manifestations due to the last two mechanisms. The increased percentage of tumor necrosis supports this aspect, as does its association with increased peritumoral edema.

Diabetes mellitus and hypertension represent comorbidities that negatively influence patient survival. Over time, the involvement of diabetes mellitus in brain tumor pathology has been controversial. While some studies have observed that varying glycemic thresholds (>112 mg/dL, >174 mg/dL, or >180 mg/dL) are associated with decreased survival, others have not found any associations [23,24]. A particularly important aspect is the effect of medication against type 2 diabetes. Metformin inhibits mechanistic targets of rapamycin (mTOR) signals by stimulating apoptosis and autophagy. The proteins p21 and p53 are responsible for these effects, which in turn lead to the destruction of cancer stem cells and the prevention of angiogenesis. The association of metformin with temozolomide leads to the inhibition of chemoresistance by inhibiting proliferation and stimulating apoptosis [25,26].

The exact mechanism by which hypertension maintains and promotes astrocytoma is unknown. The only suspected aspect is the effect of antihypertensive treatment. Amines and amides present in loop and thiazide diuretics have carcinogenic effects on the central nervous system [27]. The mechanism of sustained chronic inflammation, with impact on tumor progression and diminishing therapeutic effects, maintained by arterial hypertension and other metabolic syndromes, was noted by Aboubechara et al., but in the case of grade 4 gliomas, IDH wildtype [28].

Topographically, most tumors are supratentorial with more frequent involvement of the frontal, temporal, and parietal lobes [29]. Involvement of the midline or at least three lobes results in decreased overall survival [30]. The distribution was similar in the present study, with a higher frequency in the temporal lobe than the frontal. We did not have cases involving the midline or three lobes. In our cases, tumor location was associated only with symptomatology.

Tumor dimensions are particularly important, both in terms of the development of the processes responsible for clinical manifestations and in terms of the therapeutic approach. The most commonly used methods to quantify tumor dimensions consist of assessing the axial diameter, tumor surface area, and tumor volume. The latter can be assessed by three-dimensional ellipsoid or semi-automatic segmentation methods. The three-dimensional measurement method by semi-automatic segmentation has the highest accuracy, but does not show associations with other important tumor data [31,32]. The therapeutic measure most affected by the accuracy of the measurements is radiotherapy. The procedure requires a clear demarcation from adjacent structures (optic nerve, optic chiasm, brainstem, etc.), as well as peritumoral edema. However, volume quantification by imaging techniques is more useful for low-grade gliomas, because they do not show extensive lesions in the adjacent parenchyma and intratumoral necrosis [31,32,33].

We observed that the maximum axial diameter of tumors presents a threshold value for the development of peritumoral edema. Knowledge of these aspects leads to a greater possibility of managing intracranial hypertension and, implicitly, the symptoms produced by it [34]. Fang et al. noted that a low edema index has positive effects on progression-free survival, without correlating with patient survival [35].

A notable aspect identified by Raj et al. is the increased survival of patients in cases with large tumor volume. The explanation for this phenomenon is the hospitalization of patients in large academic hospitals, where the resectability rate is high [36]. Unlike their study, where there is no differentiation according to IDH status, our study can be considered the first to observe this phenomenon in the case of IDH mutant grade 4 gliomas.

Current surgical treatment provides gross total resection as the most appropriate tool for increasing progression-free survival and overall survival [37,38]. Some studies also admit supramaximal resection as having a positive impact on both parameters. However, it should be mentioned that this type of resection was analyzed on grade 4 gliomas regardless of IDH status [39]. It looks like a mean residual volume of less than 5 cm^3^ and a resectability rate of 70% can extend survival by 3.9 months [40]. We observed a higher resectability rate, but also a larger residual volume. These aspects highlight the importance of the resectability rate as a protective factor, as well as the presence of tumor residue and a residual volume as negative prognostic factors, which led to a high mortality rate.

According to the European Association of Neuro-Oncology guidelines, surgical ablation is followed by radiotherapy and chemotherapy. Radiotherapy begins after 3–5 weeks. 1.8–2 Gy per day is administered up to a dose of 50–60 Gy and must cover a 15 mm margin around the tumor volume. Chemotherapy is based on alkylating agents—temozolomide. Carmustine, lomustine, nimustine, and fotemustine can be used as reserve alkylating agents [41,42]. Over half of the patients in the study benefited from the complete trimodal treatment: neurosurgery, radiotherapy, and administration of temozolomide. However, the mortality rate was very high. Thus, we can say that in addition to clinical and imaging aspects, histo-molecular characteristics have a major influence on patient survival.

Hypoxia is caused by insufficient nutrient supply. This situation occurs when the partial pressure of O_2_ drops to 5–9 mmHg. With hypoxia, an acidic environment with a pH of 6.8 is created. These aspects lead to a metabolism through anaerobic glycolysis that favors and maintains the acidic pH. In this acidic and hypoxic microclimate, hypoxia-inducible factor-1 (HIF-1) causes the activation of proangiogenic genes. The consequence is neovascularization. This process entails the formation of a new vascular network and vascular regression as a result of tumor cells aggregating around preexisting vessels. Necrosis is initiated. Consequently, necrotic phenomena occur as a consequence of the newly formed vessels’ inability to sustain the demand for oxygen and nutrients [5,43,44,45]. Exposure of tumor cells to such conditions involves a metabolic adaptation and a canonical upregulation of stem cell genes (CD133, Nestin, SOX2). Metabolic adaptation consists of increasing glycolytic activity and inhibiting oxygen consumption—and so little following neovascularization processes [45].

In these types of tumors, the majority population is represented by glial stem cells. This status confers on them an increased capacity for survival and multiplication. To highlight these cells, we used nestin. It manages to provide information regarding the dedifferentiated status of the cells, the degree of malignancy, and the invasive potential [46,47]. In the present study, we noted an increased proliferative index in the case of mature cells (nestin negative/weak positive). We observed the same increased proliferative index in cells with MGMT > 50%, even with a threshold value. Furthermore, the same status was correlated with the mature cell population present. This trio of associations explains, on one hand, the metabolic changes that glial tumor cells undergo to become mature cells capable of proliferation and unresponsive to deoxyribonucleic acid (DNA) repair mechanisms, and on the other hand, the high mortality rate due to a low response to chemotherapy.

DNA methylation status is critical in therapeutic resistance. Under treatment with alkylating agents (temozolomide), MGMT removes the methyl group from O6-methylguanine, neutralizing the effects of the medication on DNA, with a consequent reduction in therapeutic efficacy. Therefore, MGMT status is considered a marker of temozolomide resistance [48,49]. Studies have shown that immunohistochemical interpretation can be an alternative surrogate for MGMT detection by polymerase chain reaction (PCR). A methylated status is considered to be a negative expression or below 10%. The consequence of this status is to increase patient survival [49,50]. In our cases, in addition to its independent factor of death, the unmethylated status of the gene was correlated with increased tumor necrosis and microvascular density.

We have noted the association between necrosis and microvascular density, but also the effect that these exert on the adjacent brain parenchyma through peritumoral edema. Necrosis stimulates angiogenesis by forming an immature and dysfunctional vascular network [25,51]. Over time, the process of angiogenesis leads to a reduction in the expression of junctional adhesion proteins and pericyte coating. These changes lead to increased leakage, with degradation of the extracellular matrix and remodeling of the microclimate [52,53]. This fragility is responsible for the collapse of the blood–brain barrier, which is characterized by the appearance of peritumoral edema [25,51]. Our study strengthens this hypothesis by identifying threshold values for predicting the appearance of peritumoral edema, both for necrosis and for microvascular density.

In the present study, we have shown the implications of microvascular density in clinical and imaging characteristics. A particular aspect is the association of the female sex with increased microvascular density. The explanation of this phenomenon is the identification of an increased expression of estrogen receptor 1 in tumors with rich vascularization. This receptor is associated with angiogenesis processes through its involvement in VEGF signaling pathways, pericyte migration, and cell adhesion. Hiller-Vallina et al. observed in grade 4 gliomas (glioblastoma IDH wildtype and astrocytoma IDH mutant) a difference in necroinflammatory and vascular processes between the sexes. In the case of the male sex, they noted the predominance of necroinflammatory effects on fragile vessels, while in groups of men and women with Estrogen Receptor 1 (ESR1) overexpression, they identified the absence of vascular fragility. These aspects strengthen the role of the receptor in the maintenance and consolidation of tumor angiogenesis [54]. From an imaging point of view, astrocytomas IDH mutant show less abundant microvascularization than the IDH-wildtype homologue. It should be mentioned that the increased density in glioblastomas, also proven at the transcriptomic level, leads to a rapid progression of the tumor towards a fatal event. In the case of astrocytomas, latency in time leads to an increase in size [55,56]. This observation aligns with our own findings.

Another mechanism derived from the hypoxic environment, with vascular implications, that can determine and maintain tumor necrosis is related to the PTEN gene. Loss of PTEN expression causes thrombotic phenomena and is associated with necrosis in high-grade gliomas [57,58]. Normally, through the PI3K-Akt-PTEN pathway, it modulates angiogenic activity and the stromal response to angiogenic stimuli. Therefore, deletion of the gene, or a loss of reaction prevents the activity of suppressing angiogenesis [59]. This study reinforces the role of PTEN in necrosis and microvascularization by highlighting the identified associations and the discovered threshold values.

An important role of the PTEN gene lies in its ability to inhibit the PI3K pathway with a protective role against p53 degradation. The PI3K-Akt pathway promotes the translocation of Murine Double Minute 2 (MDM2) from the cytoplasm to the nucleus, where it inhibits p53. In turn, p53 induces the MDM2 gene, with protein production, blocking the additional recruitment of factors necessary for the induction of gene expression (p53 modulates the activity of cells to stress and injury by activating genes involved in DNA repair, cell cycle control, angiogenesis, senescence, and apoptosis). Thus, the p53-MDM2 complex is formed and reaches the cytoplasm. Here, MDM2 inhibits the transcriptional activity of p53, causes its degradation, and prevents apoptosis. A feedback loop is created through which p53 upregulates MDM2 expression, and MDM2 downregulates p53, depending on the needs. PTEN intervenes in this pathway by blocking MDM2 at the cytoplasmic level, with its degradation and preservation of p53 function [60,61,62]. The DNA methylation status intervenes in this loop. Normally, MGMT expression is downregulated in p53 upregulation, and vice versa. In the initial stages of gliomagenesis, p53 is stabilized. With the progression of the process, MGMT expression becomes increasingly downregulated by the methylation promoter, leading to an abnormal upregulation of p53, which will induce mutations. A wild-type amount of p53 will try to combat the effect. Given that methylation is an irreversible process, MGMT upregulation will no longer occur, and mutant p53 will be the majority [63]. These interactions translate into a low survival of patients due to tumor progression [60,63]. We also identified in the present study this aspect of reduced survival in p53 overexpression and PTEN loss, in addition to their associations with reduced resectability rate and increased residual volume.

Alternate Reading Frame (ARF) plays another role in regulating p53 activity by inactivating MDM2. ARF is a tumor suppressor protein encoded by the CDKN2A gene [64]. Gene deletion not only classifies the entity but is also involved in tumor prognosis, progression-free survival, and overall survival [64,65,66,67]. We observed that these aspects related to patient survival, along with the association with microvascular density and necrotic percentage, support the involvement of gene alteration in prognosis. These data validate the findings of Appay et al.’s prospective study, which identified gene deletion as a negative risk factor for overall survival based on tumor microvascularization, regardless of whether tumor necrosis is present or not [68]. In addition, we highlighted an association between CDKN2A gene deletion and unmethylated DNA status by immunohistochemical examination, an aspect not found in the specialized literature.

Limitations of this study are represented by its retrospective nature, the fact that DNA methylation status analysis could not be performed using DNA extraction or PCR amplification, and the need for a larger group of patients. Also, the lack of molecular tests (IDH, PTEN, p53), data on patient medication, lack of external validation for thresholds, and performance scores (Karnofsky and Eastern Cooperative Oncology Group performance status) represent limitations of the study. AUC provides a single summary statistic of the discriminatory power of the model across all possible classification thresholds. However, it does not explicitly indicate performance at a specific, predefined optimal threshold relevant to clinical decision-making, which may make direct application difficult. Therefore, external validation of data on a larger sample is required. In exchange, the strengths of this study consist of the identification of risk factors, clinical-imaging, and morphogenetic factors in the survival of patients with IDH-mutant astrocytoma. Additionally, the study identifies threshold values for peritumoral edema, gene methylation status, PTEN loss, p53 overexpression, and CDKN2A gene alteration, which are primarily based on the effects of hypoxic events, necrosis, and microvascularization. This information provides important data and improves the literature, poor after the latest WHO classification, on new-old grade 4 glioma with IDH mutations. All threshold values must be integrated into future studies for their validation, preferably on much larger batches.

## 5. Conclusions

Through the present study, we were able to evaluate the consequences of hypoxic events in conjunction with clinical and morphogenetic imaging parameters, in an exploratory manner that requires external validation on larger groups. We observed that necrosis and increased microvascular density represent central elements in the pathogenic process that induce or are related to other essential changes promoting tumor aggressiveness. In addition, they are independently associated with poor prognosis and survival of patients, representing independent negative risk factors. The implication of these phenomena is associated with other changes such as p53 overexpression, PTEN loss, and CDKN2A gene mutations. To these are added the advanced age of the patients, the presence of comorbidities (diabetes mellitus and hypertension), a low resectability rate, tumor residue, and an increased tumor volume.

The results obtained may be useful in improving patient management. Imaging techniques can visualize foci of microvascularization and/or necrosis, improving neurosurgical techniques by more accurately evaluating the type of total or extended approach, possibly applying an adjuvant treatment. The results could also represent a direction for future research that would consider antiangiogenic therapies and other associations of histopathological aspects with molecular changes.

## Figures and Tables

**Table 1:** Univariate and multivariate analyses of imaging risk factors.

Parameter	Univariate Analysis			Multivariate Analysis		
	Hazard Ratio	*p* Value	Confidence Interval (CI95)	Hazard Ratio	*p* Value	Confidence Interval (CI95)
Hemisphere	1.581	0.158	0.838–2.984	1.676	0.166	0.807–3.482
Lobar topography	1.051	0.331	0.950–1.163	0.923	0.251	0.804–1.059
Peritumoral edema	1.473	0.522	0.450–4.825	4.002	0.105	0.748–21.408
Midline shift	0.998	0.952	0.948–1.051	0.916	0.061	0.835–1.004
Maximum diameter	0.997	0.765	0.979–1.015	1.018	0.491	0.967–1.072
Tumor volume	0.997	0.179	0.994–1.001	0.976	0.002	0.961–0.991
Resectibility rate	0.866	<0.001	0.817–0.917	0.801	<0.001	0.722–0.889
Residual tumor	2.662	0.022	1.150–6.160	23.473	<0.001	4.414–124.840
Residual volume	1.060	<0.001	1.034–1.087	1.153	<0.001	1.065–1.249

**Table 2:** Histopathological and genetic aspects of the study group.

Parameter	Value
Ki67 (%)	
- Average, (min–max) - Median, (IQR)	48.75, (15–90) 47.5, (25–72.5)
MGMT (%)	
- <10% - 10–50% - >50%	29.55 34.09 36.36
Nestin (%)	
- High/Medium Intensity - Low/Negative Intensity	68.18 31.82
P53 overexpressed (%)	79.55
PTEN retained (%)	72.73
Necrosis (%)	
- Average, (min–max) - Median, (IQR)	34.23, (10–69) 32, (21.5–45.25)
Microvascular density (vessels/mm^2^)	
- Average, (min–max) - Median, (IQR)	49.95, (22.6–70.8) 51.7, (37.75–63.48)
CDKN2A (%)	
- Deletion - Amplification	45.45 6.82

**Table 3:** Univariate and multivariate analyses of morphogenetic risk factors.

Parameter	Univariate Analysis			Multivariate Analysis		
	Hazard Ratio	*p* Value	Confidence Interval (CI95)	Hazard Ratio	*p* Value	Confidence Interval (CI95)
Ki67	0.999	0.829	0.987–1.011	0.950	0.005	0.916–0.984
MGMT						
- <10%	Ref.			Ref.		
- 10–50%	2.355	0.038	1.050–5.280	2.400	0.177	0.673–8.560
- >50%	1.671	0.212	0.747–3.739	2.231	0.436	0.296–16.802
Nestin—high/medium intensity	1.313	0.576	0.506–3.404	1.964	0.276	0.583–6.610
P53 overexpressed	6.962	0.002	2.035–23.820	5.114	0.038	1.094–53.897
PTEN lost	4.153	<0.001	2.113–8.162	3.658	3.658	1.198–11.173
Necrosis	1.097	<0.001	1.063–1.132	1.096	0.001	1.038–1.158
Microvascular density	1.096	<0.001	1.057–1.137	1.071	0.035	1.005–1.141
CDKN2A						
- Normal	Ref.			Ref.		
- Deletion	2.612	0.009	1.278–5.339	2.941	0.051	0.645–5.860
- Amplification	6.793	0.005	1.757–26.271	2.130	0.291	0.289–15.688

## Data Availability

The original contributions presented in this study are included in the article. Further inquiries can be directed to the corresponding author.
